# Effect of Equine-assisted Biographical Work (EABW) in older adults with subclinical depression: a randomized controlled trial

**DOI:** 10.1186/s12906-026-05315-4

**Published:** 2026-02-28

**Authors:** Julia Schmidt, Andrea Wartenberg-Demand, Simon Forstmeier

**Affiliations:** 1 Psychology and Clinical Psychology of the Lifespan, Department of Psychology, University of Siegen, Adolf-Reichwein-Str. 2a, Siegen, 57068 Germany; 2 Reittherapie Wittgenstein, Bad Berleburg, Germany; 3 Pferdegestützte Interventionen, Nidda, Germany

**Keywords:** Equine-assisted therapy, Prevention, Subclinical depression, Anxiety, Quality of Life, Randomized controlled trial

## Abstract

**Introduction:**

Preventive approaches for depression in adults aged 50 years and older have received little attention, despite increasing needs arising from demographic changes. In particular, subclinical depression is often underdiagnosed and associated with anxiety, a poorer quality of life, and greater need for assistance from the healthcare system.

**Objective:**

This multicentre, randomised, controlled phase III trial investigated if Equine-Assisted Biographical Work (EABW) is effective to improve subclinical depression.

**Methods:**

Qualified sites in Germany enrolled 52 participants ≥ 50 years (Full Analysis Set (FAS), *N* = 50). The intervention received weekly sessions of EABW for 8 weeks. The control group was not treated. Follow-up was after 3 months. Participants in both groups had similar demographic and baseline characteristics.

The primary endpoint was change in Beck Depression Inventory-II (BDI-II). Secondary target parameters included questionnaires, e.g., dealing with anxiety, behavioural avoidance, and state of health.

**Results:**

The pre-test BDI-II score averaged 20.92 (SD 5.99) in the intervention group and 22.36 (SD 6.80) in the control group with a highly significantly greater improvement after treatment (F = 15.21, *p* < 0.001, Cohen’s d = 1.80) for intervention group. This difference was maintained until the follow-up period (F = 15.23, *p* < 0.001, d = 1.43). This outcome was supported by an improvement of secondary endpoints at week 8: Beck Anxiety Inventory (F = 8.38, *p* < 0.001, d = 0.93), Ego-Integrity Questionnaire (F = 19.60, *p* < 0.001, d = 1.32), Questionnaire for Positive and Negative Spontaneous Thoughts (F = 9.95, *p* < 0.001, d = 1.07), Gratitude Questionnaire (F = 12.71, *p* < 0.001, d = 1.09), Cognitive Behavioural Avoidance Scale (F = 18.26, *p* < 0.001, d = 1.50), Reminiscence Functions Scale (identity, F = 7.93, *p* < 0.001, d = 0.93; problem-solving, F = 5.88, *p* = 0.004, d = 0.85), Short Form Health Survey (36) (mental, F = 8.41, *p* < 0.001, d = 1.11), General Self-Efficacy Scale (F = 9.44, *p* < 0.001, d = 0.89), Multidimensional Mood State Questionnaire. For the Short Form Health Survey (36) (physical, F = 0.17, *p* = 0.84, d = 0.11) and other subgroups of the Reminiscence Functions Scale (death preparation, bitterness revival, boredom reduction, intimacy, conversation, and teach) no differences were observed.

**Conclusions:**

EABW was able to achieve a significant and lasting treatment success. This approach may represent an innovative concept to prevent worsening of depressive symptoms, improve anxiety and quality of life. The gentle use of the horse as a medium turned out to be very effective in achieving therapeutic results and acted as a kind of gateway for therapeutic processes.

**Trial registration:**

German Clinical Trials Register DRKS00017010. Registered on 01 April 2019.

**Supplementary Information:**

The online version contains supplementary material available at 10.1186/s12906-026-05315-4.

## Introduction

This clinical trial investigated adults from the aged 50 years and older with subclinical depression.

According to Guineau and colleagues (2023) a different symptom network of psychological functioning is observed above the age of 50. Even though the World Health Organization describes older adults as adults aged 60 and above, there is no clear consensus in the literature about an appropriate age cut-off [16]. Therefore, we decided to include a population with a cut-off at 50 years and older to prevent the development of a major depression. This population is often impacted by changes in living conditions, underdiagnosed with regard to subclinical depression and associated with a poorer quality of life and greater need for assistance from the healthcare system [6, 15].

This phase of life is characterized by significant emotional, social, motor, and cognitive alterations. Additionally, people in this age group often experience critical life events, such as the loss of loved ones, changes in the workplace, and the onset of diseases [2]. In light of the ongoing demographic change, interventions for older adults become even more important for improving mental health.

Biographical work and life review interventions could be especially beneficial for this age population, as research showed that older adults often feel the need to look back on their lives for a balanced life review [7]. The risk of subclinical depression progressing to a major depression, one of the most common mental disorders in older adults, is increased [27]. A meta-analysis has found small-to-moderate effects for psychotherapy (primarily cognitive-behavioural therapy) for subclinical depression [8]. In contrast, studies that utilize structured biographical work or reminiscence interventions to enhance general well-being in individuals with depression show moderate-to-large effects [4, 25]. Furthermore, a meta-analysis has reported a large effect for reminiscence interventions in the treatment of clinical depression in older adults, with a standardized mean difference (g = 1.09) [24].

Equine-assisted Biographical Work (EABW; German: Pferdegestützte Biografiearbeit – Erwachsene, PBA-E) [29] is a concept combining elements of equine-assisted intervention [26, 32] and biographical work or life review [17]. Biographically important topics are addressed systematically, considering the different phases of life from child- to adulthood, using the horse as a medium. The therapeutic approach is based on a human-animal bond. Through the horse's body language and its species-specific behavior, the conventional therapeutic setting is shifted from a dyad between therapist and client into a triadic relationship, with the horse's resonance is integrated into the process. This innovative approach has been successfully applied in various case studies [29]. This innovative concept unfolds new opportunities in treating older adults with subclinical depression.

This multicentre randomised controlled trial (RCT) was designed to demonstrated whether a preventive, equine-assisted, age-specific treatment with EABW is more effective than no intervention in treating individuals with subclinical depression, using the BDI-II as primary endpoint supported by a variety of secondary endpoints [29].

## Materials and methods

The EABW phase III trial was conducted at 11 qualified sites in Germany. It was performed in accordance to the International Council for Harmonisation (ICH), Good Clinical Practice (GCP) standards, the Declaration of Helsinki, and with the approval of an independent ethics committee. All participants provided written informed consent. The first participant was enrolled on July 06, 2019, and the last one left the trial on October 07, 2020. The publication of the trial protocol can be found in the Supplement 2.

### Participants

Participants were adults ≥ 50 years old with symptoms of subclinical depression defined by the following inclusion and exclusion criteria [29]:

Inclusion criteria:

1. Age ≥ 50.
2. Symptoms of subclinical depression (BDI-II ≥ 9).
3. Sufficient physical and mental resilience.
4. Adequate language skills.
5. Written informed consent.

Exclusion criteria.

1. Equinophobia (fear of horses).
2. Severe horse hair allergy.
3. Diagnosis of major depression based on SCID-5-CV.
4. Acute suicidal tendency.
5. Psychotic disorders known from anamnesis.
6. Known dementia.
7. Known severe systemic diseases (e.g., cancer and Parkinson’s disease).
8. Intake of chemical or herbal antidepressants.
9. Participation in psychotherapeutic treatment 4 weeks before or during the study (including follow up).
10. Participation in another study within the last 30 days before inclusion.
11. Employment by the therapist.
12. Familial relation to the therapist.

The BDI-II and SCID-5-CV were taken before trial inclusion. Other parameters were taken from the medical records and interview with the participants.

Sample size estimations controlled for type I error to 5% with statistical power of 0.8. Therefore, with a medium effect size, α = 0.05, a test strength of 1-*β* = 0.80 and a correlation between repeated measures of *r* = 0.6, the sample size resulted in an n of 42 [12]. Assuming a drop-out rate of 20%, a total sample size resulted in *n* = 52 (i.e., 26 subjects in each group).

### Procedure

Each trial centre was responsible for recruiting participants. The therapists received informational recruitment material approved by the ethics committee. Potential participants underwent a screening procedure, and those who met all incclusion criteria were randomised into the trial. The allocation ratio for randomisation into either the intervention group (IG) or in the control group (CG) was 1:1. A randomisation list was created using the software RandList (http://randomisation.eu) before the study began [29]. The study coordinator received individual allocations via encoded email. Informed consent was obtained before the screening procedure started.

The main investigator at each site served as a responsible contact person and was familiar with all the guidelines of the professional association for equine-assisted intervention in Germany, as well as the "Quality assurance for equine-assisted interventions" guidelines [21].

The following criteria had been fulfilled to qualify as a therapist:

- an educational, psychological, therapeutic or medical profession,
- profound equestrian qualification,
- recognised education in the field of equine-assisted intervention approved by the professional association “Berufsverband für Fachkräfte Pferdegestützter Interventionen e.V.”the first and after the last session, participants of the IG were interviewed about their psychological and health status. Short questionnaires were used before and after the sessions to demonstrate the present psychological state of the participants. A follow-up survey was raised 3 months after the last intervention
- completion in a one-day training course on EABW at the study site (initiation visit) and trainings conducting a study according to the International Council for Harmonisation—Good Clinical Practice (ICH-GCP) guidelines.

The central medium in EABW was the specially trained horse. The sites were inspected during regular monitoring visits by a qualified monitor.

Directly before the first and after the last session, participants of the IG were interviewed about their psychological and health status. Short questionnaires were used before and after the sessions to demonstrate the present psychological state of the participants. A follow-up survey was raised 3 months after the last intervention. Participants assigned to the CG received no intervention while using the same questionnaires at randomisation and 8 weeks later. All participants performed the follow-up test after 3 months.

Part of the study took place during the COVID-19 pandemic, which did not significantly affect study conduct. Deviations were documented accordingly. During this time relevant guidelines were continuously checked and applied, monitoring visits were partly conducted remotely by telephone rather than on site, as travel options were limited. In addition, a risk assessment protocol was prepared by the study committee, and relevant factors were regularly reviewed.

All study documents were filed in a Trial Master File (TMF), quality controls were performed and the TMF was archived at the end of the study.

To demonstrate clinically meaningful effects, guided interventions were offered and extensively trained. Furthermore, validated and recognized questionnaires were used. Standard Operation Procedures (SOPs) were adhered to, including regular monitoring visits for quality purposes.

### Assessment

The primary and secondary endpoints were measured using a clinical questionnaire battery. Validated self-evaluation questionnaires were used to test effectiveness. The participants had sufficient time to complete the evaluation questionnaires without being influenced by the professionals; questions were restricted to clarifications about completing the questionnaires. Additionally, the theoretical basis for the development of each instrument is provided, along with detailed interpretation guidelines and information on the quality criteria for the questionnaires used.

### Primary outcome

The primary outcome is the improvement of subclinical depression after eight weeks of treatment, measured by the BDI-II. The BDI-II is a self-assessment questionnaire that determines depressive symptoms by using 21 items on a four-point Likert scale. Higher scores are associated with more severe levels of depression. The questionnaire asks about the mental state of the study participant during the last 14 days including up to the survey date [3].

### Secondary outcome

The secondary endpoints included the following questionnaires: Beck Anxiety Inventory (BAI), Ego-Integrity Questionnaire (EI), Questionnaire for Positive and Negative Spontaneous Thoughts (FAG), Gratitude Questionnaire (GQ-6), Cognitive Behavioural Avoidance Scale (CBAS), Reminiscence Functions Scale (RFS-28), Short Form Health Survey (36) (SF-36), General Self-Efficacy Scale (GSE), and Multidimensional Mood State Questionnaire (MDMQ). All questionnaires are completed in printed form.

The Beck Anxiety Inventory is a self-assessment questionnaire to measure the severity of anxiety and consists of 21 items. The items relate to physiological and cognitive aspects of anxiety. Using a four-point scale, the study participant has the opportunity to describe their subjective feelings over the last seven days.

The Ego-Integrity Questionnaire is a self-assessment scale that measures personality development in old age. The self-assessment is based on 16 items (e.g., “If I had to do it all over again, there are very few things in my life that I would change.”/“I am still angry about certain childhood experiences.”), using a six-point scale.

The Questionnaire for Positive and Negative Spontaneous Thoughts consists of 21 items, with three assigned scales (Negative self-statements, Well-being, Self-confidence). Using a five-point scale, the participant evaluates his or her thoughts over the past week.

The Gratitude Questionnaire is a self-assessment scale that measures the subjective experience of gratitude in everyday life. The questionnaire contains six items (e.g., “I am grateful for many blessings in my life”) and uses a seven-point numerical intensity scale with verbal description.

The Cognitive-Behavioral Avoidance Scale includes four subscales which measure behavioral social (BS) avoidance, behavioral non-social (BN) avoidance, cognitive social (CS) avoidance, and cognitive non-social (CN) avoidance and 31 items (e.g., “I avoid interacting with other people when I am feeling low or anxious”). The statements are assessed on a five-point Likert scale.

The Reminiscence Function Scale determines why and when people remember their past. The evaluation is carried out on a six-point numerical intensity scale (Identity, Intimacy Maintenance, Conversation, Boredom Reduction, Teach/Inform, Problem Solving, Death Preparation, Bitterness Revival).

The Short Form Health Survey measures health-related quality of life and can be used in different diseases. It consists of 36 items (e.g., “How would you describe your state of health in general?”).

The General Self-Efficacy Scale is used as a self-assessment procedure to record general optimistic self-beliefs. The questionnaire contains a one-dimensional scale with 10 items (e.g., “I can usually handle whatever comes in my way”).

The Multidimensional Mood State Questionnaire is a self-assessment instrument for recording the current psychological well-being. The MDMQ short form is made up of 12 items (e.g., At this moment I feel: … “rested”, “tired”, “unwell”).

Citations and additional exploratory parameters not shown herein are provided in the publication of the study protocol in Supplement 2.

### Treatments

Trial participants in the IG received once a week 90-min sessions of EABW over a period of 8 weeks. Participants were seen by the therapist during regular visits. The sessions of EABW followed a standardised concept, with two sessions on childhood, two on adolescence, three on adulthood and one for the integration of memories from different life phases (Table 1) [29]. The developmental phases of the individual stages of life are worked out with specific tasks and the inclusion of the horse to initiate the therapeutic process [30]. Through interaction with the horse, your own patterns of actions and behaviors are immediately reflected back, allowing new realms of experience to emerge.

**Table 1:** Description of sessions

No	Unit	Focus	Topic
1	Childhood I	Habitat capture	- Exploration of the horse's habitat - Triggering of childhood memories - Possible use of nostalgic objects
2	Childhood II	Relationship work	- Relationship building with the horse through body-oriented work - Exploration of the horse's body - Use of a massage technique - Preparation of the lifeline part 1
3	Adolescence I	Constellation task	- Demonstration of relationships with the help of the horse - Use of horse figures as representatives for family relationships
4	Adolescence II	Communication	- Observing and naming the non-verbal communication of horses - Naming of characteristics of difficult human communication - Preparation of the lifeline part 2
5	Young adulthood	Self-assertion training	- Experiences and dealing with stress - Guiding rope work - Positioning
6	Adulthood I	Parcourse work	- Experiencing and accepting challenges - Dealing with obstacles, formulation of objectives
7	Adulthood II	Mindfulness training	- Recognizing and naming one's own needs - Preparation of the lifeline part 3
8	Integration	Gain in knowledge	- Collecting findings from the previous units - Response to open questions - Review of the complete lifeline

The manual includes, in addition to a thematic introduction, a wide variety of templates and materials in the form of documents, worksheets, and therapeutic questions. The therapeutic questions have been adapted from Rybarczyk & Bellg (1997). These questions pertain to childhood and adolescence (e.g., What strengths and weaknesses did your parents have? Did you grow up in the city or in the countryside? Did you have any pets? What positive values and ideals did your parents instill in you?) as well as adulthood (e.g., What was the most enriching experience of your life? What was your proudest moment? What was the greatest challenge you had to face in your life?). The special aspect of horse-assisted biographical work is that parts of the therapeutic conversation already take place during the intervention with the horse, thereby facilitating an easier entry and access to emotional topics. More details of the intervention can be found in Supplement 2.

### Statistical analysis

All statistical methods were prespecified in the study protocol and the statistical analysis plan.

Efficacy analyses were performed in the Full Analysis Set (FAS) which included all randomised participants who received one intervention and underwent at least one BDI-II testing after baseline.

Safety analysis used the Safety Analysis Set (SAS) which comprised all participants who received at least one intervention.

All statistical evaluations were done with IBM SPSS Statistics 25. A two-sided significance level of 5% was applied to all tests. A two-factor analysis of covariance (ANCOVA) was calculated with group as between-factor and time as within-factor (repeated measures) and the covariates age, sex, and concomitant diseases.

Due to the pandemic an addendum to the statistical analysis plan was prepared. The influence of the pandemic was calculated for the primary and secondary target parameters, with no significant influence on the results was found. There were also no relevant anomalies in the safety parameters [29].

## Results

### Participant flow and characteristics of the sample

**Fig. 1:**
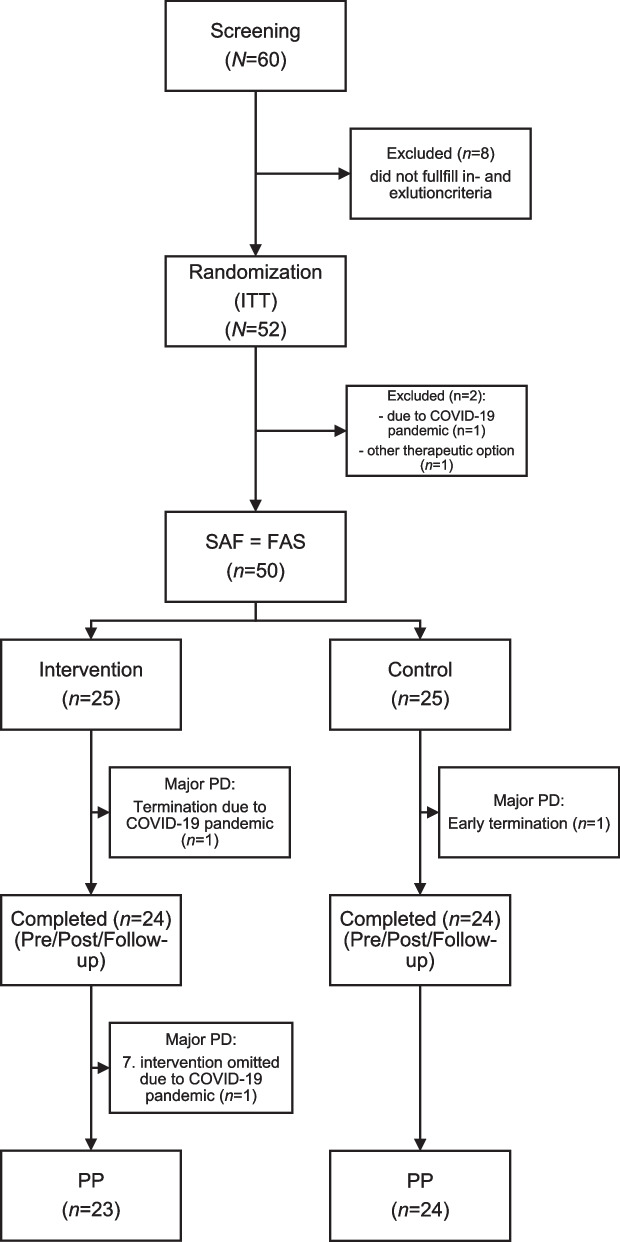
Participants` disposition

Out of 60 subjects screened, a total of 50 participants (45 female [90%] and 5 males [10%]; mean [SD] age, 57.06 [4.86] years) were enrolled from July 06, 2019 through October 07, 2021 (Fig. 1). 48 participants completed the trial. Including all participants, the BDI-II scores averaged at 20.92 (13.00–34.00) for the pre-test data in the IG and 22.36 (11.00–39.00) in the CG while not fulfilling the criteria of major depression according to a SCID-5-CV structured interview. No significant differences in baseline characteristics were observed including socioeconomic status such as education and marital status (Table 2). Only 5 participants (10%) had previous experience with equine assisted therapy.

**Table 2:** Demographics and other baseline characteristics

	**Intervention** ***n* = 25**	**Control** ***n* = 25**	**Total** ***N* = 50**	**Sig** (2-sided)
Age at screening				0,313
Mean (SD)	56,36 (4,67)	57,76 (5,04)	57,06 (4,86)	
Median	56,00	57,00	57,00	
Min—Max	50—65	50—66	50—66	
Gender, n (%)				0,157
Male	1 (4%)	4 (16%)	5 (10%)	
Female	24 (96%)	21(84%)	45 (90%)	
Education, n (%)				0,089
Vocational training	19 (76%)	13 (52%)	32 (64%)	
University degree	6 (24%)	12 (48%)	18 (36%)	
Marital status, n (%)				0,113
Married	16 (64%)	13 (52%)	29 (58%)	
Divorced	1 (4%)	6 (24%)	6 (24%)	
Widowed	1 (4%)	1 (4%)	1 (4%)	
Single	7 (28%)	3 (12%)	3 (12%)	
In relationship	0	2 (8%)	2 (8%)	
Retirement status, n (%)				0,480
Retired	4 (16%)	6 (24%)	10 (20%)	
Not retired	21 (84%)	19 (76%)	40 (80%)	
Beck depression inventory (BDI-II) Pretest				0,431
Mean (SD)	20,92 (5,99)	22,36 (6,80)	21,64 (6,39)	
Median	19,00	21,00	20,50	
Min—Max	13,00—34,00	11,00—39,00	11,00—39,00	
Number previous diseases participants (%)	11 (44,00%)	17 (68,00%)	28 (56,00%)	0,087
Previous diseases, n				0,092
Mean (SD)	0,68 (0,95)	1,16 (1,03)	0,92 (1,01)	
Median	0,00	1,00	1,00	
Min—Max	0—3	0—3	0—3	
Number concomitant diseases participants (%)	13 (52%)	16 (64%)	29 (58%)	0,390
Concomitant diseases, n				0,726
Mean (SD)	1,36 (2,58)	1,16 (1,18)	1,26 (1,99)	
Median	1,00	1,00	1,00	
Min—Max	0—12	0—5	0—12	
Number concomitant medication participants (%)	12 (48%)	13 (52%)	25 (50%)	0,777
Concomitant medication, n				0,899
Mean (SD)	0,84 (1,25)	0,80 (0,96)	0,82 (1,10)	
Median	0,00	1,00	0,50	
Min—Max	0—5	0—3	0—5	

### Primary outcome

The primary outcome (BDI-II in pre-post comparison, week 1 to week 8) was met in the full analysis set (*N* = 50) with a highly significant time x group interaction (F = 15.21, *p* < 0.001) and a large between effect size (Cohen’s d = 1.80). In the IG the BDI-II score (week 1, pre-test) was 20.92 (SD = 5.99) and dropped to a value of 7.32 (SD = 6.21) under therapy, while in the CG the pre-post comparison changed only from 22.36 (SD = 6.80) to 20.48 (SD = 9.71) (Table 3). This evaluation was supported by the per-protocol set (*N* = 47) (IG, pre-test: M = 21.30, SD = 6.02/post-test, M = 7.21, SD = 6.39; CG, pre-test: M = 22.83, SD = 6.51/post-test M = 21.12, SD = 9.35; *p* < 0.001). The influence of COVID-19 pandemic was evaluated and showed no difference (F = 1.22, *p* = 0.28).

**Table 3:** Results on the Course of Depression (BDI-II) Effect Sizes, and Condition x Time Interaction (FAS)

	**Pretest**		**Posttest**		**Follow-up**		**d**_**within**_		**Interaction** **(group x time)**		**d**_**between**_	
	**M**	**SD**	**M**	**SD**	**M**	**SD**	**Pre-Post**	**Pre-FU**	**F**	**p**	**Pre-Post**	**Pre-FU**
IG (*n* = 25)	20,92	5,99	7,32	6,21	8,80	8,13	2,23	1,70	15,23	< 0,001***	1,80	1,43
CG (*n* = 25)	22,36	6,80	20,48	9,71	19,56	13,30	0,22	0,27				
Total (*N* = 50)	21,64	6,39	13,90	10,45	14,18	12,19						

A two-factor ANOVA was calculated with repeated measures and the factors group and time as well as the covariates age, sex, and concomitant diseases

*IG* Intervention group, *CG* Control group, *M* Mean, *SD* Standard deviation, *F* Statistical parameter, *p* significance value, *d* Cohen's d, *Pre* Pretest, *Post* Posttest, *FU* Follow-up, Intervention group (IG), control group (CG)

^*^p < 0,05, **p < 0,01, ***p < 0,001

**Fig. 2:**
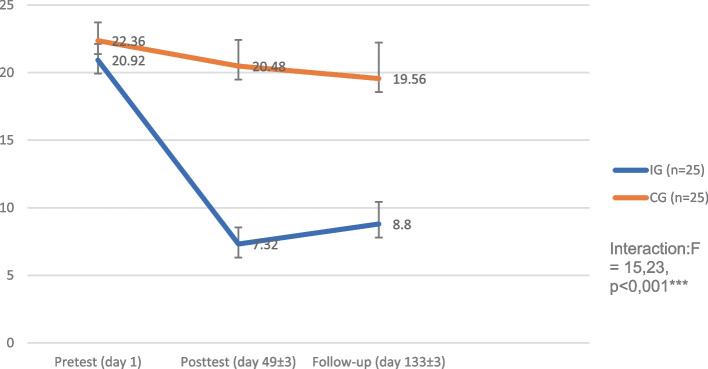
Depression symptoms (BDI-II) over time per condition

The course of the BDI-II from pre-test to follow-up is shown in Fig. 2. Again, the BDI-II showed a significant result in favour of the IG (interaction: F = 15.23, *p* < 0.001; see Tab. 3) and a large between effect size (Cohen’s d = 1.43) during the course until the follow-up survey at week 20. The BDI-II improved systematically from visit to visit in the IG during the course of therapy. At the follow-up examination, the BDI-II value was 8.80 for the IG and 19.56 for the CG.

### Secondary outcomes

For the secondary endpoints significant results were found in almost all parameters with psychological dimensions (as shown at post-test and follow-up). The results should be interpreted in an exploratory sense since the study was not powered for the secondary endpoints. Especially, the Beck Anxiety Inventory (F = 8.38, *p* < 0.001, Cohen’s d = 0.93/0.85), Cognitive Behavioural Avoidance Scale (F = 18.26, *p* < 0,001, Cohen’s d = 1.50/1.16), Ego-Integrity Questionnaire (F = 16.9, *p* < 0.001, Cohen’s d = 1.32/0.92), Questionnaire for Positive and Negative Spontaneous Thoughts (F = 9.95, *p* < 0.001, Cohen’s d = 1.07/0.86), Gratitude Questionnaire (F = 12.71, *p* < 0.001, Cohen’s d = 1.09/0.56), General Self-Efficacy Scale (F = 9.44, *p* < 0.001, Cohen’s d = 0.89/0.69), and in the three subcategories of the Multidimensional Mood State Questionnaire depicted significant results in pre-post comparison.

For the Reminiscence Functions Scale only the dimensions identity (F = 7.93, *p* < 0.001, Cohen’s d = 0.93/0.11) and problem solving (F = 5.88, *p* < 0.004, Cohen’s d = 0.85/0.40) were significant. The Short Form Health Survey (36) showed a significant improvement in the quality of life in the mental dimension (F = 8.41, *p* < 0.001, Cohen’s d = 1.11/0.73). This could not be confirmed for the physical dimension (F = 0.17, *p* = 0.84, Cohen’s d = 0.18/0.07). The results of all secondary outcomes are provided in detail in Table 4 (Supplement 1).

### Adverse events

A total of 45 adverse events occurred in the trial. Five were documented as serious adverse events (SAE) and 40 as adverse events (AE). Of the 50 participants enrolled in the trial, adverse events (AE/SAE) occurred in 25 participants (50%). In the CG, 4 SAEs and 14 AEs occurred, and in the IG, 1 SAE and 26 AEs occurred. Slightly more AEs occurred in the IG, which can be explained by regular questioning and external influences. None of the AEs/SAEs had a causal relationship to treatment. No participant died during the trial.

## Discussion

This RCT, which included 50 participants, is the first to examine EABW in adults over the age of 50 years with subclinical depression. The trial demonstrated a significant clinically meaningful and stronger reduction in depressive symptoms for the intervention group compared to the control group by applying the BDI-II.

Although the symptoms of subclinical depression are less severe than having a major depression it is associated with a great health service burden. Improving mental health in this population is considered medically important and represents a promising target for preventive and earlier interventions for developing major depression. Subclinical depression is often underdiagnosed and a risk factor for developing major depression. Moreover, existing literature show that the estimated prevalence varies greatly.

The prevalence of anxiety symptoms is 67% in patients with subclinical depression [5]. Therefore, not only depressive symptoms, but also anxiety symptoms could be significantly reduced at follow-up. In addition, the EABW had a significant positive impact on ego integrity, a key aspect of personality development in old age. This means that dealing with one's own life biography is already relevant from the age of 50 in order to initiate health-promoting processes, among other things [18].

All dimensions of positive and negative automatic thoughts (i.e., negative self-statements, self-confidence, well-being) showed a significant result in favour of the intervention group. Thus, EABW positively stimulates the inner dialogue leading to a resource-strengthening attitude.

There is evidence that the feeling of gratitude is associated with greater psychological and physical well-being and, consequently, a low feeling of gratitude is negatively related to emotional disorders such as depression and social anxiety [20, 22]. It makes sense that gratitude can also be significantly increased in the intervention group as compared to the control group.

The Cognitive-Behavioural Avoidance Scale identifies clinically relevant avoidance behaviour with particular relevance for depressive avoidance behaviour. CBAS scores also correlate with depression and anxiety scores [28], which was confirmed for the intervention group with significant results.

The Reminiscence Function Scale showed significant result for the intervention group for the dimensions identity and problem solving, which are the most important positive reminiscence styles that were intended to be changed. However, no significant results were obtained for the dimensions preparation for death, embitterment revival, boredom reduction, intimacy, conversation and teaching.

The effectiveness of the intervention was further assessed by examining quality of life using the SF-36. While the mental health subscore increased significantly more in the intervention group compared to the control group, this was not the case for the physical subscore. The physical dimensions corresponded to those of a normal population and did not change under therapy. However, it should be noted, that participants were between 50 and 66 years old and had few relevant comorbidities [10, 11]. Consequently, apart from the psychological problems, the trial involved a relatively physically healthy population in the second half of life.

Self-Efficacy, i.e., the individual assessment of one's own competencies to cope with difficulties and obstacles by one's own efforts [19], also increased significantly more in the intervention group compared to the control group.

### Clinical considerations

EABW is a preventive concept that combines elements of equine-assisted interventions and biographical work. By taking a structured look back at one's own life story, individual resources can be recognized and used to shape the future. The developmental phases of the individual stages of life are taken into account as intervention goals through specific tasks with the horse. The gentle use of the horse as a medium has proven highly effective in facilitating therapeutic results, serving as a gateway to therapeutic processes.

In an additional survey regarding the therapies´ value, participants were able to provide information through questionnaires and free text responses. The resonance of including a horse in the therapeutic approach was overwhelmingly positive.

Cuijpers and colleagues (2014) reported in a meta-analysis that in subclinical depression the effects of psychological treatments are not as pronounced as in the treatment of major depression [8]. For example, a clinical trial comparing cognitive-behavioural therapy (CBT, *n* = 20) with treatment as usual (TAU, *n* = 23) over a period of 7 weeks (follow-up at 3 and 6 months) in older participants with subclinical depression (completed study with all assessments group) revealed a significant effect on depressive symptoms in favour of the treatment group [23]. A further clinical trial showed similar results of a treatment called Coping with Depression (CWD) course [1]. However, in the EABW, even more significant effects were achieved by combining biographical work with equine-assisted therapy.

Healthy ageing is a holistic process that can be shaped across all phases of life. Ensuring both physical and mental health is therefore important for individuals and society at every age. Health promotion and prevention should therefore be prioritized across the lifespan [31].

Psychotherapeutic care for the older population appears to be inadequate, which may be attributed to factors such as the stigma surrounding mental illness, fears of (self-)stigmatization and care shortages in both urban and rural areas [14, 33].

Our results suggest that the preventive and resource-oriented concept of EABW could serve as an additional therapeutic option for improving psychological symptoms and mental health in this population. In consequence, conducting larger clinical trials with robust study designs is proposed [9].

### Strengths and limitations

This RCT is the first to evaluate equine-assisted biographical work with several important contributions to the literature on subclinical depression. First, the large effect sizes for depression and moderate or large effect sizes for secondary outcomes are larger compared to other psychotherapeutic approaches [8]. Second, the treatment was manualized, the study therapists received intensive training and continuing supervision based on the session checklists, securing treatment fidelity. Third, the sample size was based on a power calculation using a moderate effect size that was exceeded by the actual results. Fourth, the trial was conducted according to the criteria of Good Clinical Practice with a high scientific standard, which is still an exception in the field of equine assisted interventions. A strict design was followed with a comprehensive oversight and monitoring of the clinical trial.

The results should be interpreted in the light of several limitations. First, follow-up was at three months posttreatment; longer follow-up intervals could demonstrate more convincingly the long-term effect of the treatment. Second, we did not apply a dismantling design, e.g., a treatment group receiving biographical work alone and a group receiving equine-assisted therapy alone, in order to allow conclusions about the efficacy of treatment components. Third, the efficacy results of EABW cannot be generalized to samples of older patients in general age because of the age range of 50–66 years. Future studies should use a sample of individuals age 65 years and older. Forth, the predominance of female participants may limit the generalizability of the results. The trial included a higher proportion of women, consistent with epidemiological data showing a greater prevalence of depression in this age group. Previous research also indicates that women are more likely to engage in equine-assisted therapy, which may explain the observed gender distribution. This pattern aligns with meta-analyses on depression that similarly report a predominance of female participants. Finally, while some socioeconomic factors (e.g., education, marital status, retirement status) were collected, others such as income and health behaviors (e.g., smoking, physical activity, diet) were not. Since these variables can influence both depressive symptoms and treatment outcomes [13], their omission represents a limitation of this trial. Nevertheless, for the socioeconomic factors that were assessed, no statistically significant differences emerged between the two study groups.

## Conclusions

The EABW trial examined the efficacy and safety of equine-assisted biography work in adults (≥ 50 years) with subclinical depression in an 8-week intervention followed by a 3-month follow-up period. A control group without intervention served as a comparison. The well-being of the participants, which was assessed by validated questionnaires, showed significant and sustained improvements in both the primary outcome variable (BDI-II) and the secondary outcome variables. The interventions were found to be safe. No causal relationship was documented for any of the adverse events. In addition, a survey of participants on the value of the therapy was overwhelmingly positive.

## Supplementary Information

Supplementary Material 1.

Supplementary Material 2.

Supplementary Material 3.

## Data Availability

Data are available from the corresponding author upon request with a signed data access agreement.

## Abbreviations

AE: Adverse event
ANCOVA: Analysis of covariance
ANOVA: Analysis of variance
BAI: Beck Anxiety Inventory
BDI-II: Beck Depression Inventory – II
CBAS: Cognitive Behavioural Avoidance Scale
CBT: Cognitive-behavioural therapy
CG: Control group
CWD: Coping with Depression
DoH: Declaration of Helsinki
DSM-V: Diagnostic and Statistical Manual of Mental Disorders
EABW: Equine-assisted biographical work
EI: Ego-Integrity Questionnaire
FAG: Questionnaire for Positive and Negative Spontaneous Thoughts
FAS: Full Analysis Set
GCP: Good Clinical Practice
GQ-6: Gratitude Questionnaire
GSE: General Self-Efficacy Scale
ICH: International Council for Harmonisation
IG: Intervention group
MDMQ: Multidimensional Mood State Questionnaire
PBA-E: Pferdegestützte Biografiearbeit—Erwachsene
PP: Per Protocol Set
RCT: Randomised controlled trial
RFS- 28: Reminiscence Functions Scale
SAE: Serious adverse event
SAS: Safety Analysis Set
SCID-5-CV: Structured Clinical Interview for DSM-5® Disorders—Clinical Version
SD: Standard Deviation
SF-36: Short Form Health Survey (36)
SOPs: Standard Operation Procedures
TAU: Treatment as usual
TMF: Trial Master File
