# Supplementary material for: Effect of Equine-assisted Biographical Work (EABW) in older adults with subclinical depression: a randomized controlled trial
Source: BMC Complement Med Ther. 2026 Feb 28;26:124. doi: 10.1186/s12906-026-05315-4 (PMC13059425; doi:10.1186/s12906-026-05315-4)
Supplement: Supplementary file 1 — Supplementary Material 1. [file 12906_2026_5315_MOESM1_ESM.docx]

**Supplement 1**

Table 4. Results on the Course of Beck Anxiety Inventory (BAI), Ego-Integrity Questionnaire (EI), Questionnaire for Positive and Negative Spontaneous Thoughts (FAG), Gratitude Questionnaire (GQ-6), Cognitive Behavioral Avoidance Scale (CBAS), Reminiscence Functions Scale (RFS-28), Short Form Health Survey (SF-36), and General Self-Efficacy Scale (GSE) in Full Analysis Set (FAS)

**Table 4. Results on the Course of Beck Anxiety Inventory (BAI), Ego-Integrity Questionnaire (EI), Questionnaire for Positive and Negative Spontaneous Thoughts (FAG), Gratitude Questionnaire (GQ-6), Cognitive Behavioral Avoidance Scale (CBAS), Reminiscence Functions Scale (RFS-28), Short Form Health Survey (SF-36), and General Self-Efficacy Scale (GSE) in Full Analysis Set (FAS).**

|  |  |  | **Pretest** | | | **Posttest** | | | **Follow-up** | | | **Interaction**  **(group x time)** | | **Effect size** | | |
| --- | --- | --- | --- | --- | --- | --- | --- | --- | --- | --- | --- | --- | --- | --- | --- | --- |
|  |  |  | **M** | **SD** | | **M** | **SD** | | **M** | **SD** | | **F** | **p** |  | **Post** | **FU** |
| **BAI** | Beck Anxiety Inventory | IG | 34,40 | | 6,89 | 28,52 | | 5,97 | 28,92 | | 6,65 | 8,38 | <0,001*** | d (IG Pre-Post/FU): | 0,91 | 0,81 |
|  |  | CG | 36,75 | | 6,55 | 37,21 | | 8,67 | 37,04 | | 9,66 |  |  | d (IG vs. CG): | 0,93 | 0,85 |
|  |  | total | 35,57 | | 6,76 | 32,87 | | 8,58 | 32,98 | | 9,18 |  |  |  |  |  |
| **EI** | Ego-Integrity Questionnaire | IG | 4,05 | | 0,64 | 4,87 | | 0,57 | 4,67 | | 0,72 | 19,60 | <0,001*** | d (IG Pre-Post/FU): | 1,35 | 0,91 |
|  |  | CG | 3,92 | | 0,55 | 3,94 | | 0,71 | 3,98 | | 0,74 |  |  | d (IG vs. CG): | 1,32 | 0,92 |
|  |  | total | 3,99 | | 0,60 | 4,40 | | 0,79 | 4,33 | | 0,80 |  |  |  |  |  |
| **FAG** | Negative self-statements | IG | 2,40 | | 0,75 | 1,66 | | 0,48 | 1,72 | | 0,72 | 9,95 | <0,001*** | d (IG Pre-Post/FU): | 1,18 | 0,93 |
|  |  | CG | 2,31 | | 0,61 | 2,31 | | 0,76 | 2,23 | | 0,81 |  |  | d (IG vs. CG): | 1,07 | 0,86 |
|  |  | total | 2,36 | | 0,68 | 1,98 | | 0,71 | 1,98 | | 0,80 |  |  |  |  |  |
| **FAG** | Self-confidence | IG | 2,34 | | 0,87 | 3,32 | | 0,85 | 2,76 | | 0,97 | 13,18 | <0,001*** | d (IG Pre-Post/FU): | 1,14 | 0,46 |
|  |  | CG | 2,36 | | 0,81 | 2,33 | | 0,84 | 2,38 | | 1,06 |  |  | d (IG vs. CG): | 1,18 | 0,47 |
|  |  | total | 2,35 | | 0,83 | 2,83 | | 0,98 | 2,57 | | 1,02 |  |  |  |  |  |
| **FAG** | Well-being | IG | 2,04 | | 0,80 | 3,10 | | 0,83 | 2,89 | | 0,93 | 13,61 | <0,001*** | d (IG Pre-Post/FU): | 1,30 | 0,98 |
|  |  | CG | 2,22 | | 0,50 | 2,16 | | 0,82 | 2,28 | | 0,91 |  |  | d (IG vs. CG): | 1,65 | 1,17 |
|  |  | total | 2,13 | | 0,67 | 2,63 | | 0,95 | 2,58 | | 0,96 |  |  |  |  |  |
| **GQ-6** | Gratitude | IG | 31,80 | | 5,66 | 37,52 | | 4,48 | 35,48 | | 4,97 | 12,71 | <0,001*** | d (IG Pre-Post/FU): | 1,12 | 0,69 |
|  |  | CG | 32,40 | | 5,76 | 31,80 | | 6,48 | 32,64 | | 6,20 |  |  | d (IG vs. CG): | 1,09 | 0,59 |
|  |  | total | 32,10 | | 5,66 | 34,66 | | 6,23 | 34,06 | | 5,74 |  |  |  |  |  |
| **CBAS** | Total | IG | 2,64 | | 0,47 | 2,11 | | 0,47 | 2,17 | | 0,46 | 18,26 | <0,001*** | d (IG Pre-Post/FU): | 1,13 | 1,01 |
|  |  | CG | 2,57 | | 0,41 | 2,71 | | 0,59 | 2,62 | | 0,56 |  |  | d (IG vs. CG): | 1,50 | 1,16 |
|  |  | total | 2,61 | | 0,44 | 2,41 | | 0,61 | 2,40 | | 0,55 |  |  |  |  |  |
| **CBAS** | Behavioral, non-social avoidance | IG | 2,92 | | 0,48 | 2,38 | | 0,67 | 2,43 | | 0,57 | 11,61 | <0,001*** | d (IG Pre-Post/FU): | 0,93 | 0,93 |
|  |  | CG | 2,78 | | 0,65 | 2,92 | | 0,65 | 2,89 | | 0,58 |  |  | d (IG vs. CG): | 1,17 | 1,03 |
|  |  | total | 2,85 | | 0,57 | 2,65 | | 0,71 | 2,66 | | 0,62 |  |  |  |  |  |
| **CBAS** | Behavioral, social avoidance | IG | 2,44 | | 0,73 | 1,95 | | 0,70 | 1,98 | | 0,66 | 9,90 | <0,001*** | d (IG Pre-Post/FU): | 0,69 | 0,66 |
|  |  | CG | 2,53 | | 0,73 | 2,71 | | 0,91 | 2,48 | | 0,85 |  |  | d (IG vs. CG): | 0,90 | 0,55 |
|  |  | total | 2,48 | | 0,72 | 2,33 | | 0,89 | 2,23 | | 0,79 |  |  |  |  |  |
| **CBAS** | Cognitive, non-social avoidance | IG | 2,62 | | 0,53 | 2,08 | | 0,45 | 2,16 | | 0,51 | 16,24 | <0,001*** | d (IG Pre-Post/FU): | 1,10 | 0,88 |
|  |  | CG | 2,65 | | 0,51 | 2,79 | | 0,64 | 2,76 | | 0,56 |  |  | d (IG vs. CG): | 1,29 | 1,08 |
|  |  | total | 2,63 | | 0,51 | 2,43 | | 0,66 | 2,46 | | 0,61 |  |  |  |  |  |
| **CBAS** | Cognitive, social  avoidance | IG | 2,65 | | 0,63 | 2,11 | | 0,61 | 2,21 | | 0,66 | 8,89 | <0,001*** | d (IG Pre-Post/FU): | 0,87 | 0,68 |
|  |  | CG | 2,35 | | 0,57 | 2,42 | | 0,71 | 2,37 | | 0,69 |  |  | d (IG vs. CG): | 0,10 | 0,75 |
|  |  | total | 2,50 | | 0,61 | 2,27 | | 0,67 | 2,29 | | 0,67 |  |  |  |  |  |
| **RFS-28** | Identity | IG | 10,72 | | 3,91 | 13,20 | | 3,46 | 10,92 | | 3,67 | 7,93 | <0,001*** | d (IG Pre-Post/FU): | 0,67 | 0,53 |
|  |  | CG | 12,24 | | 2,47 | 11,64 | | 2,99 | 12,08 | | 3,16 |  |  | d (IG vs. CG): | 0,93 | 0,11 |
|  |  | total | 11,48 | | 3,33 | 12,42 | | 3,30 | 11,50 | | 3,44 |  |  |  |  |  |
| **RFS-28** | Problem-solving | IG | 13,92 | | 3,98 | 16,72 | | 2,94 | 14,08 | | 4,03 | 5,88 | 0,004 | d (IG Pre-Post/FU): | 0,80 | 0,04 |
|  |  | CG | 16,00 | | 3,50 | 15,56 | | 3,32 | 14,64 | | 3,71 |  |  | d (IG vs. CG): | 0,85 | 0,40 |
|  |  | total | 14,96 | | 3,85 | 16,14 | | 3,16 | 14,36 | | 3,84 |  |  |  |  |  |
| **RFS-28** | Death preparation | IG | 6,80 | | 4,01 | 7,20 | | 4,67 | 7,04 | | 4,78 | 0,45 | 0,64 | d (IG Pre-Post/FU): | 0,09 | 0,05 |
|  |  | CG | 7,88 | | 3,42 | 8,40 | | 3,73 | 8,92 | | 3,80 |  |  | d (IG vs. CG): | 0,03 | 0,21 |
|  |  | total | 7,34 | | 3,73 | 7,80 | | 4,23 | 7,98 | | 4,38 |  |  |  |  |  |
| **RFS-28** | Bitterness revival | IG | 6,68 | | 3,60 | 5,36 | | 2,27 | 6,04 | | 3,21 | 0,74 | 0,46 | d (IG Pre-Post/FU): | 0,45 | 0,19 |
|  |  | CG | 7,72 | | 2,73 | 7,36 | | 2,86 | 7,68 | | 3,63 |  |  | d (IG vs. CG): | 0,31 | 0,20 |
|  |  | total | 7,20 | | 3,21 | 6,36 | | 2,75 | 6,86 | | 3,49 |  |  |  |  |  |
| **RFS-28** | Boredom reduction | IG | 6,76 | | 3,24 | 6,08 | | 1,93 | 7,40 | | 3,87 | 0,39 | 0,68 | d (IG Pre-Post/FU): | 0,26 | 0,18 |
|  |  | CG | 8,24 | | 3,71 | 8,12 | | 3,90 | 8,12 | | 3,49 |  |  | d (IG vs. CG): | 0,16 | 0,22 |
|  |  | total | 7,50 | | 3,53 | 7,10 | | 3,21 | 7,76 | | 3,67 |  |  |  |  |  |
| **RFS-28** | Intimacy | IG | 6,16 | | 3,01 | 6,44 | | 2,18 | 6,64 | | 2,64 | 0,15 | 0,86 | d (IG Pre-Post/FU): | 0,11 | 0,17 |
|  |  | CG | 7,32 | | 3,04 | 7,32 | | 2,46 | 7,28 | | 2,87 |  |  | d (IG vs. CG): | 0,09 | 0,17 |
|  |  | total | 6,74 | | 3,05 | 6,88 | | 2,34 | 6,96 | | 2,75 |  |  |  |  |  |
| **RFS-28** | Conversation | IG | 6,68 | | 2,90 | 6,84 | | 3,05 | 6,36 | | 2,80 | 0,79 | 0,46 | d (IG Pre-Post/FU): | 0,05 | 0,11 |
|  |  | CG | 8,16 | | 2,01 | 7,48 | | 2,54 | 7,64 | | 2,40 |  |  | d (IG vs. CG): | 0,33 | 0,08 |
|  |  | total | 7,42 | | 2,58 | 7,16 | | 2,79 | 7,00 | | 2,66 |  |  |  |  |  |
| **RFS-28** | Teachings | IG | 10,92 | | 3,55 | 11,00 | | 3,30 | 10,28 | | 2,88 | 2,10 | 0,13 | d (IG Pre-Post/FU): | 0,02 | 0,20 |
|  |  | CG | 12,52 | | 3,83 | 10,68 | | 3,59 | 11,16 | | 3,93 |  |  | d (IG vs. CG): | 0,51 | 0,46 |
|  |  | total | 11,72 | | 3,74 | 10,84 | | 3,42 | 10,72 | | 3,44 |  |  |  |  |  |
| **SF-36** | State of health (mental)  State of health (physical) | IG | 34,28 | | 10,34 | 46,70 | | 10,60 | 44,59 | | 13,01 | 8,41 | <0,001*** | d (IG Pre-Post/FU): | 1,19 | 0,88 |
|  |  | CG | 33,68 | | 7,40 | 35,94 | | 9,86 | 37,36 | | 13,06 |  |  | d (IG vs. CG): | 1,11 | 0,73 |
|  |  | total | 33,98 | | 8,91 | 41,32 | | 11,49 | 40,98 | | 13,41 |  |  |  |  |  |
|  |  | IG | 51,38 | | 9,56 | 52,39 | | 8,32 | 51,41 | | 9,06 | 0,17 | 0,84 | d (IG Pre-Post/FU): | 0,11 | 0,003 |
|  |  | CG | 44,80 | | 10,17 | 44,05 | | 9,59 | 44,07 | | 11,01 |  |  | d (IG vs. CG): | 0,18 | 0,07 |
|  |  | total | 48,09 | | 10,32 | 48,22 | | 9,83 | 47,74 | | 10,64 |  |  |  |  |  |
| **GSE** | General Self-Efficacy | IG | 26,52 | | 3,47 | 30,20 | | 4,30 | 28,56 | | 4,78 | 9,44 | <0,001*** | d (IG Pre-Post/FU): | 0,94 | 0,49 |
|  |  | CG | 25,80 | | 5,02 | 25,60 | | 5,12 | 24,80 | | 5,55 |  |  | d (IG vs. CG): | 0,89 | 0,69 |
|  |  | total | 26,16 | | 4,28 | 27,90 | | 5,22 | 26,68 | | 5,46 |  |  |  |  |  |

*Notes: IG = intervention group; CG = control group; M = mean; SD = standard deviation; F = statistical parameter; p = significance value; d = Cohens d; Pre = pretest; Post = posttest; FU = follow-up; BAI = Beck Anxiety Inventory; EI = Ego-Integrity Questionnaire; FAG = Questionnaire for Positive and Negative Spontaneous Thoughts; GQ-6 = Gratitude Questionnaire; CBAS = Cognitive Behavioral Avoidance Scale; RFS-28 = Reminiscence Functions Scale; SF-36 = Short Form Health; GSE = General Self-Efficacy Scale*

*Total N = 50, intervention group (IG) = 25, control group (CG) = 25, *p<0,05, **p<0,01, ***p<0,001. d: Cohen’s d. d (IG-CG): Controlled effect size at follow-up. A two-factor ANOVA was calculated with repeated measures and the factors group and time as well as the covariates age, sex, and concomitant diseases.*
