# Supplementary material for: Effect of Equine-assisted Biographical Work (EABW) in older adults with subclinical depression: a randomized controlled trial
Source: BMC Complement Med Ther. 2026 Feb 28;26:124. doi: 10.1186/s12906-026-05315-4 (PMC13059425; doi:10.1186/s12906-026-05315-4)
Supplement: Supplementary file 3 — Supplementary Material 3. [file 12906_2026_5315_MOESM3_ESM.docx]

**Supplement 3**

Table 5. Medical History of Participants

|  | **Intervention**  ***n*=25** | | **Control**  ***n*=25** | | **Total**  ***N*=50** | |
| --- | --- | --- | --- | --- | --- | --- |
| **Study participants – medical history *n* (%)** | **11** | **(44%)** | **17** | **(68%)** | **28** | **(56%)** |
| Alcoholism | 2 | (8%) | 1 | (4%) | 3 | (6%) |
| Arterial hypertension | 0 | (0%) | 1 | (4%) | 1 | (2%) |
| Arthrosis | 1 | (4%) | 0 | (0%) | 1 | (2%) |
| Bronchial asthma | 0 | (0%) | 1 | (4%) | 1 | (2%) |
| Herniated disc | 0 | (0%) | 1 | (4%) | 1 | (2%) |
| Lyme disease | 0 | (0%) | 1 | (4%) | 1 | (2%) |
| Burnout | 1 | (4%) | 3 | (12%) | 4 | (8%) |
| Depression | 3 | (12%) | 8 | (32%) | 11 | (22%) |
| Endometriosis | 1 | (4%) | 0 | (0%) | 1 | (2%) |
| Fatigue | 0 | (0%) | 1 | (4%) | 1 | (2%) |
| Dysfunction of the gall bladder | 1 | (4%) | 0 | (0%) | 1 | (2%) |
| Hepatitis E | 1 | (4%) | 0 | (0%) | 1 | (2%) |
| Myocardial infarction | 0 | (0%) | 2 | (8%) | 2 | (4%) |
| Hyperthyroidism | 0 | (0%) | 1 | (4%) | 1 | (2%) |
| Hypothyroidism | 0 | (0%) | 1 | (4%) | 1 | (2%) |
| Multiple sclerosis | 0 | (0%) | 2 | (8%) | 2 | (4%) |
| Surgical interventions | 4 | (16%) | 5 | (20%) | 9 | (18%) |
| Insomnia | 1 | (4%) | 1 | (4%) | 2 | (4%) |
| Stroke | 1 | (4%) | 0 | (0%) | 1 | (2%) |
| Pain syndrome | 0 | (0%) | 1 | (4%) | 1 | (2%) |
| Tinnitus | 1 | (4%) | 1 | (4%) | 2 | (4%) |
| Atrial fibrillation | 1 | (4%) | 0 | (0%) | 1 | (2%) |
| *Abbreviations: N = total number of study participants; n = number of study participants*  *Note: Pearson's chi-square for frequencies and t-test for mean comparisons*  *Source data: Appendix 12.4 Statistical analyses - Table 3.1 medical history etc.xlsx* | | | | | | |

Table 6. Concomitant Diseases of Participants

|  | **Intervention**  ***n*=25** | | **Control**  ***n*=25** | | **Total**  ***N*=50** | |
| --- | --- | --- | --- | --- | --- | --- |
| **Study participants – concomitant diseases *n* (%)** | **13** | **(52%)** | **16** | **(64%)** | **29** | **(58%)** |
| Allergy | 1 | (4%) | 0 | (0%) | 1 | (2%) |
| Arterial hypertension | 2 | (8%) | 2 | (8%) | 4 | (8%) |
| Bronchial asthma | 1 | (4%) | 3 | (12%) | 4 | (8%) |
| Complaints of the skeletal system and soft tissue | 9 | (36%) | 9 | (26%) | 18 | (36%) |
| Burnout | 1 | (4%) | 0 | (0%) | 1 | (2%) |
| Chronic obstructive pulmonary lung disease (COPD) | 1 | (4%) | 0 | (0%) | 1 | (2%) |
| Anxiety | 1 | (4%) | 0 | (0%) | 1 | (2%) |
| Fatigue | 1 | (4%) | 0 | (0%) | 1 | (2%) |
| Vascular diseases | 1 | (4%) | 1 | (4%) | 2 | (4%) |
| Skin diseases | 3 | (12%) | 4 | (16%) | 7 | (14%) |
| Cardiac arrhythmia | 0 | (0%) | 1 | (4%) | 1 | (2%) |
| Infections | 2 | (8%) | 2 | (8%) | 4 | (8%) |
| Coronary heart disease | 0 | (0%) | 1 | (4%) | 1 | (2%) |
| Laboratory findings | 1 | (4%) | 0 | (0%) | 1 | (2%) |
| Migraine | 1 | (4%) | 1 | (4%) | 2 | (4%) |
| Multiple sclerosis | 1 | (4%) | 0 | (0%) | 1 | (2%) |
| Kidney disease | 0 | (0%) | 2 | (8%) | 2 | (4%) |
| Post-traumatic stress disorder (PTSD) | 1 | (4%) | 1 | (4%) | 2 | (4%) |
| Thyroid disease | 4 | (16%) | 1 | (4%) | 5 | (10%) |
| Sleep apnoea | 1 | (4%) | 0 | (0%) | 1 | (2%) |
| Tinnitus | 1 | (4%) | 0 | (0%) | 1 | (2%) |
| Menopausal symptoms | 1 | (4%) | 0 | (0%) | 1 | (2%) |
| *Abbreviations: N = total number of study participants; n = number of study participants*  *Note: Pearson's chi-square for frequencies and t-test for mean comparisons*  *Source data: Appendix 12.4 Statistical analyses - Table 3.1 Concomitant diseases etc.xlsx* | | | | | | |
